# Supplementary material for: A Proteomic Study Suggests Stress Granules as New Potential Actors in Radiation-Induced Bystander Effects
Source: Int J Mol Sci. 2021 Jul 26;22(15):7957. doi: 10.3390/ijms22157957 (PMC8347418; doi:10.3390/ijms22157957)
Supplement: Supplementary file 1 [file ijms-22-07957-s001.zip › ijms-1291943-supplementary.pdf]

Supplementary figures: statistical analysis

Sup Figure S1 Cyclophilin A (PPIA ; P62937) (Peptidyl-prolyl cis-trans isomerase A)
Sup Figure S2 Thioredoxin (TXN ; P10599)
Sup Figure S3 Alpha-enolase (ENO1 ; P06733)
Sup Figure S4 60S acidic ribosomal protein P0 (RPLP0; P05388)
Sup Figure S5 Heat shock cognate 71 kDa protein (HSC70 or HSPA8 ; P11142)
Sup Figure S6 Stress-70 protein (HSPA 9 ; P38646)
Sup Figure S7 T-complex protein 1 subunit gamma (CCT3 ; P49368)

Sup Figure S1

Cyclophilin A (PPIA ; P62937)
(Peptidyl-prolyl cis-trans isomerase A)

Results 2D Gel
SPOT 39
PPIA Fold = + 1,62

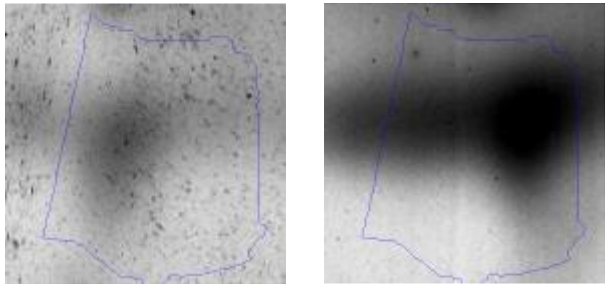

CTR 0,1

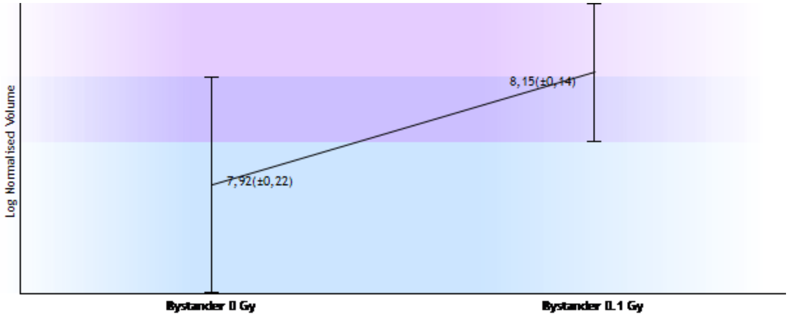

Anova
p = 0,0335

Results WB
PPIA = +37%

PPIA (18kDa)

α- Tubulin (50 Kda)

Gapdh (35 Kda)

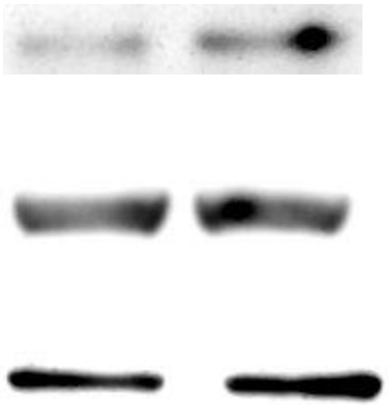

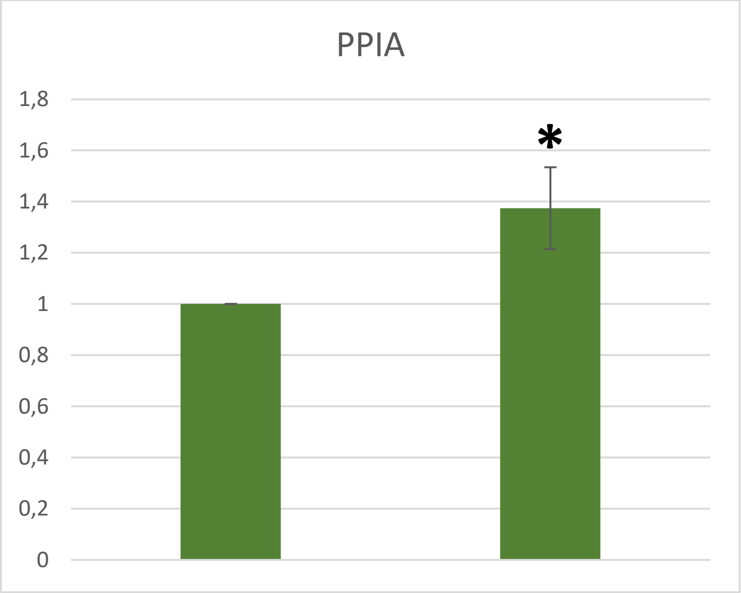

Student test
p = 0,041

Sup Figure S2

Thioredoxin (TXN ; P10599)

Results 2D Gel
SPOT 116
TXN Fold = + 1,27

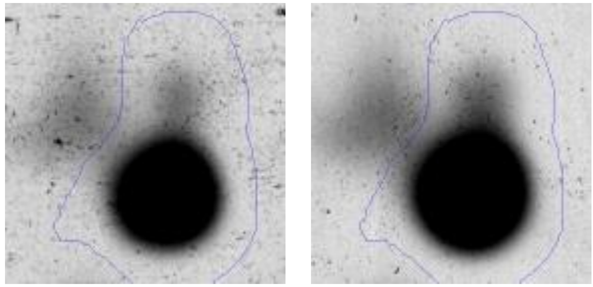

CTR   0,1

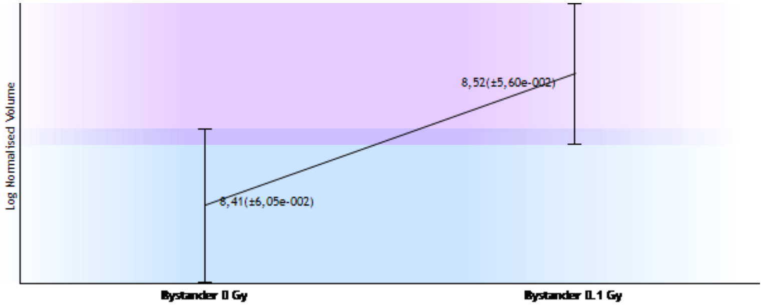

Anova
p = 0,0044

Results WB
TXN = +29%

TXN (11 Kda)

α- Tubulin (50 Kda)

Gapdh (35 Kda)

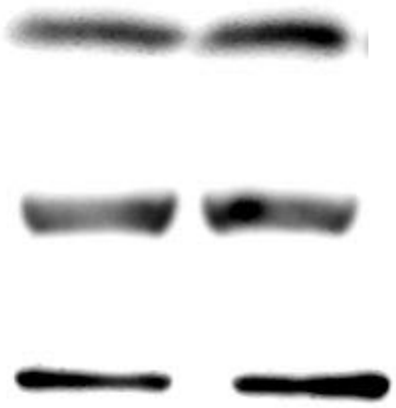

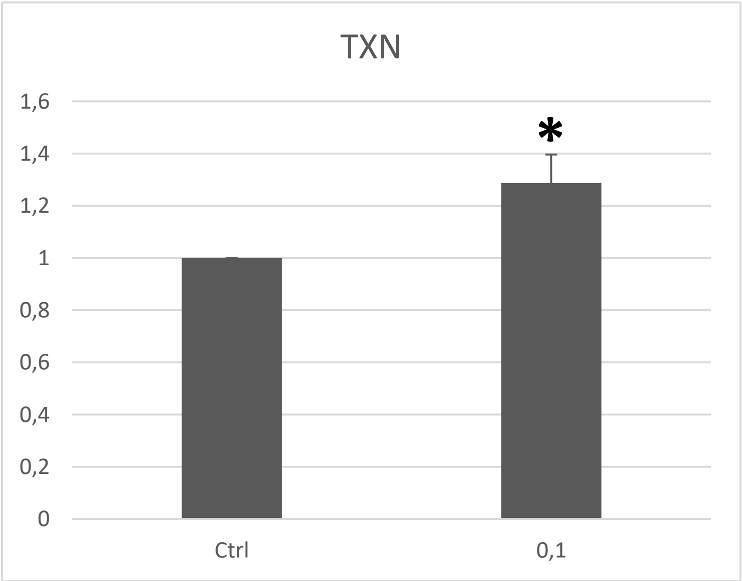

Student test
p = 0,014

Sup Figure S3

Alpha-enolase (ENO1 ; P06733)

Results 2D Gel
SPOT 65
ENO1 Fold = – 1,81

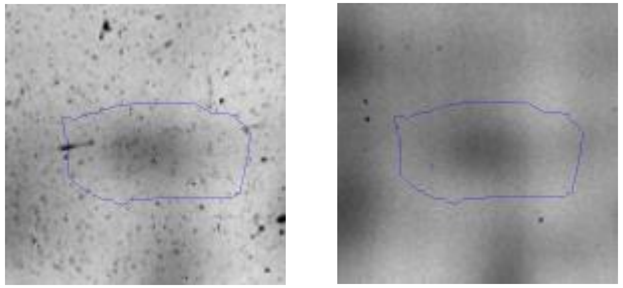

CTR   0,1

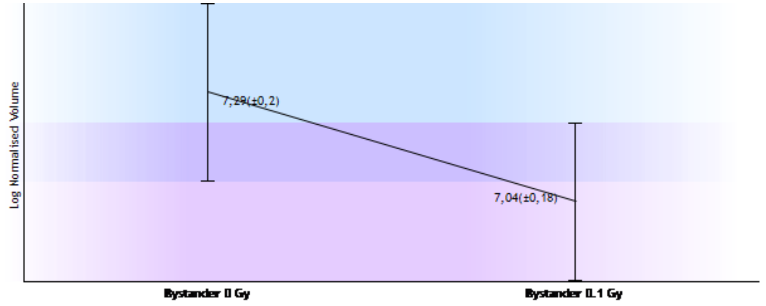

Anova
p = 0,0246

Results WB
ENO1 = -30 %

ENO1 (50 kDa)

α- Tubulin (50 Kda)

Gapdh (35 Kda)

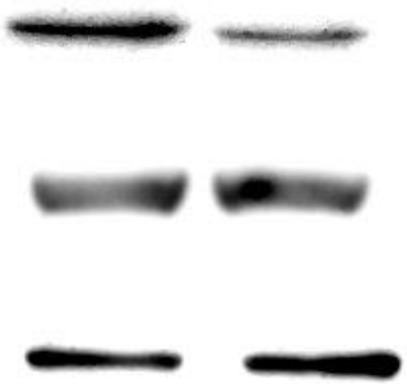

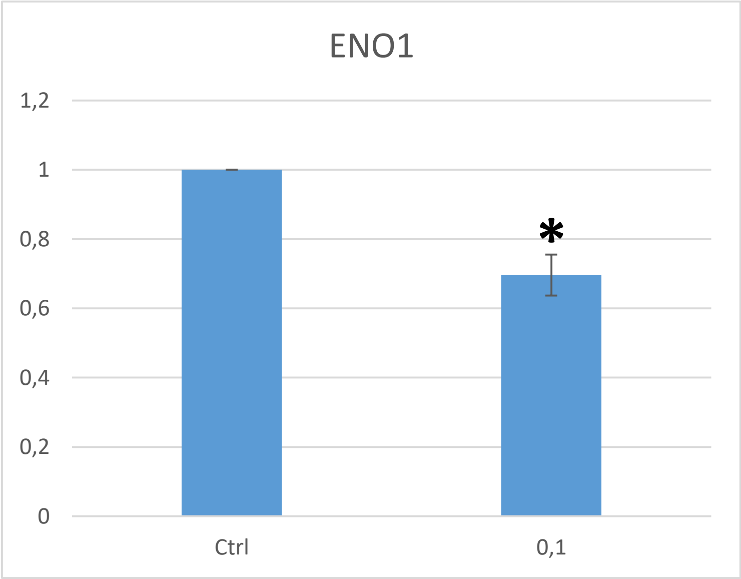

Student test
p = 0,0003

Sup Figure S4

60S acidic ribosomal protein P0 (RPLP0; P05388)

Results 2D Gel

SPOT 65

RPLP0 Fold = – 1,81

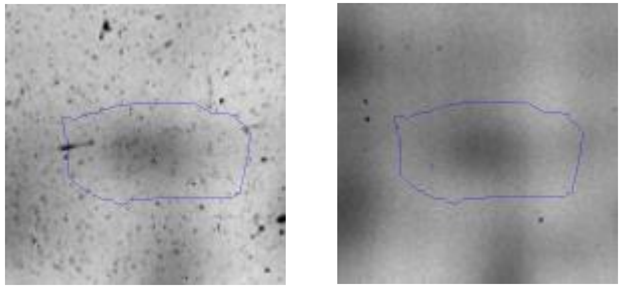

CTR 0,1

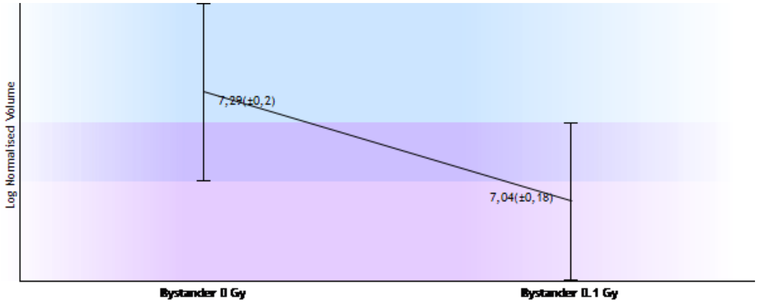

Anova
p = 0,0246

Resultats WB

RPLP0 = -18%

RPLP0 (35kDa)

α- Tubulin (50 Kda)

Gapdh (35 Kda)

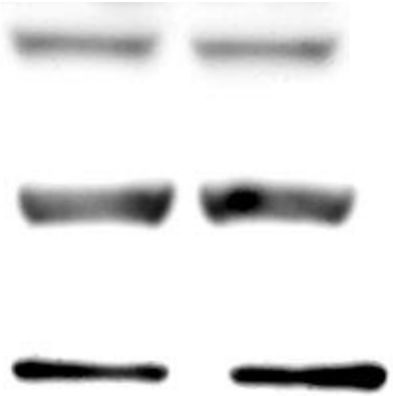

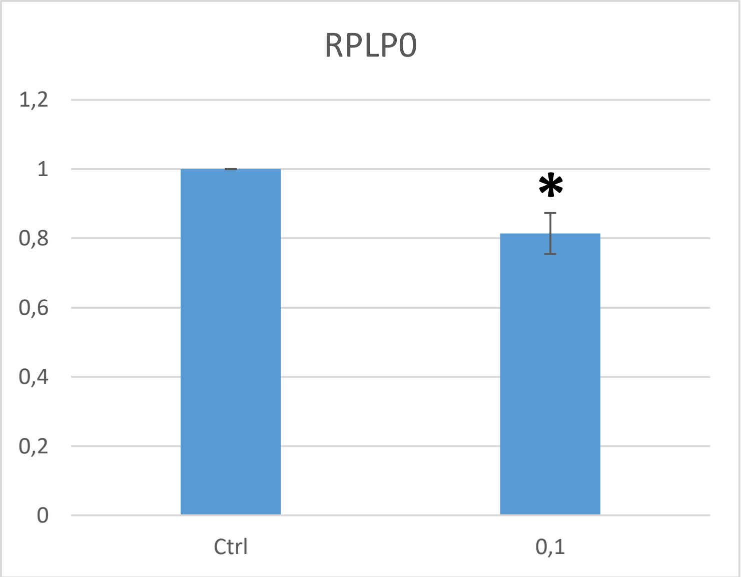

Student test
p = 0,004

Sup Figure S5

Heat shock cognate 71 kDa protein (HSC70 or HSPA8 ; P11142)

Results 2D Gel
SPOT 91
HSC70 Fold = – 1,29

Anova
p = 0,0340

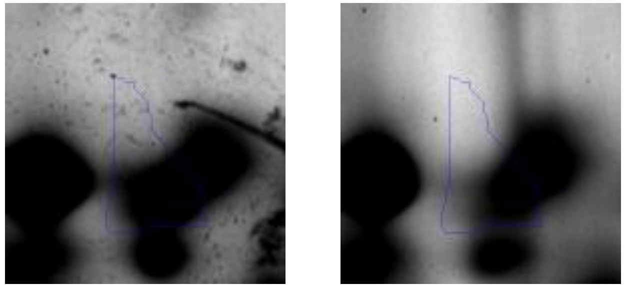

CTR 0,1

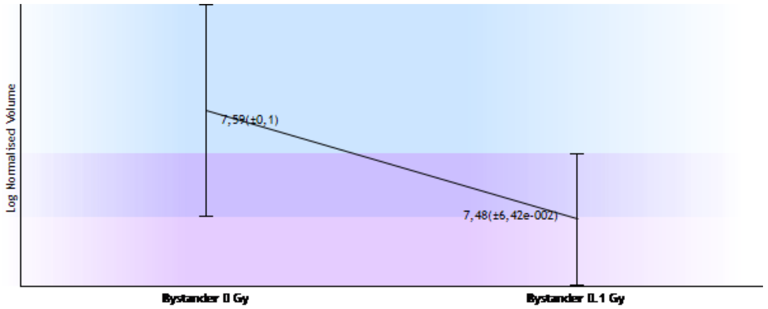

Results WB
HSC70 = -29%

HSC70 (70 kDa)
α- Tubulin (50 Kda)
Gapdh (35 Kda)

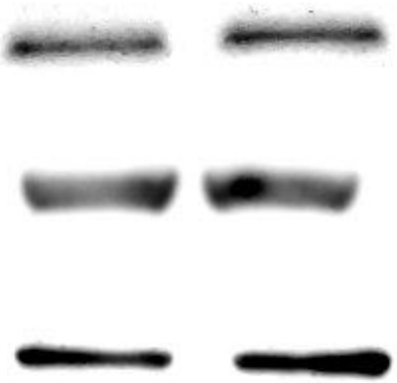

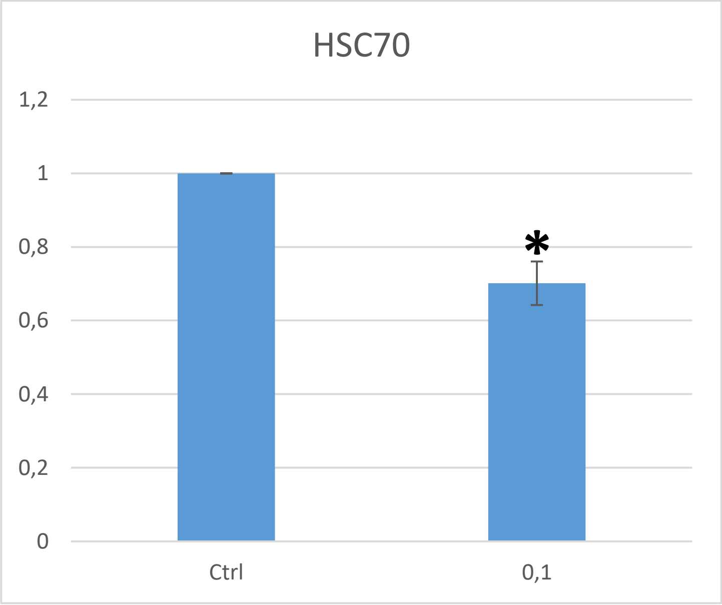

Student test
p = 0,002

Sup Figure S6

Stress-70 protein (HSPA 9 ; P38646)

Results 2D Gel
SPOT 97
HSPA Fold = – 1,32

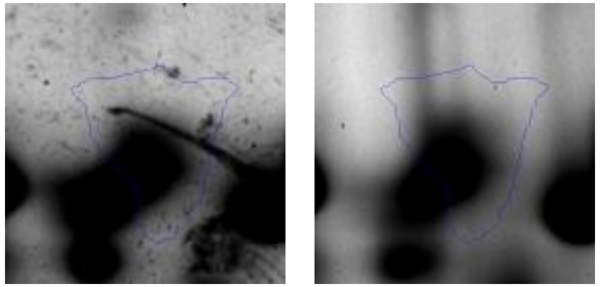

CTR 0,1

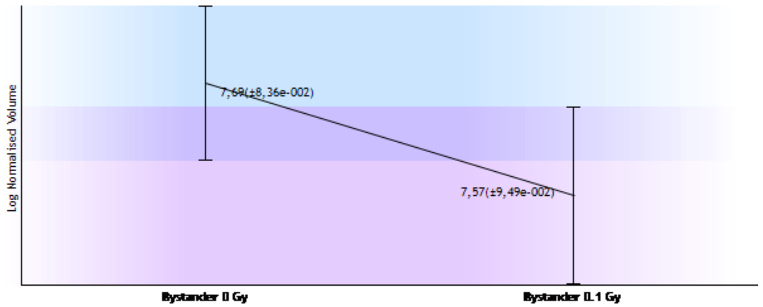

Anova
p = 0,0177

Results WB
HSPA = -26%

HSPA9 (75 kDa)
α- Tubulin (50 Kda)
Gapdh (35 Kda)

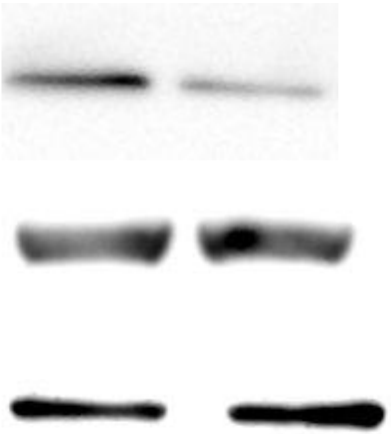

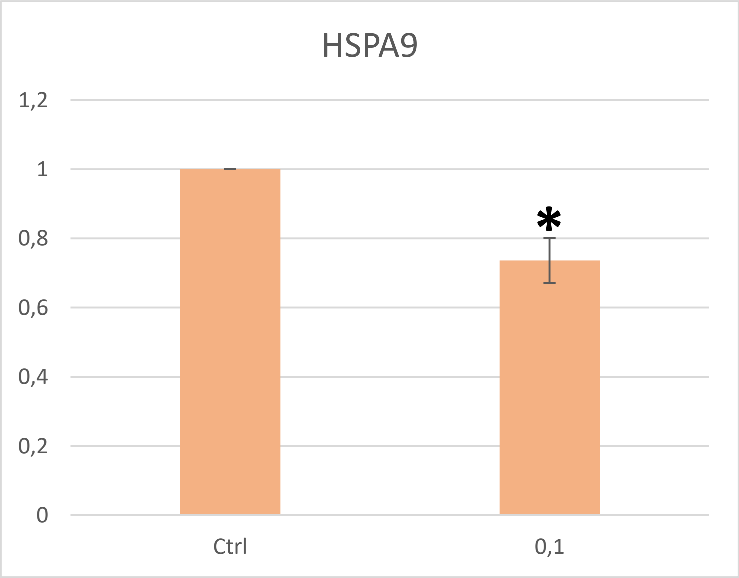

Student test
p = 0,001

Sup Figure S7

T-complex protein 1 subunit gamma (CCT3 ; P49368)

Results 2D Gel
SPOT 123
CCT3 Fold = -1,31

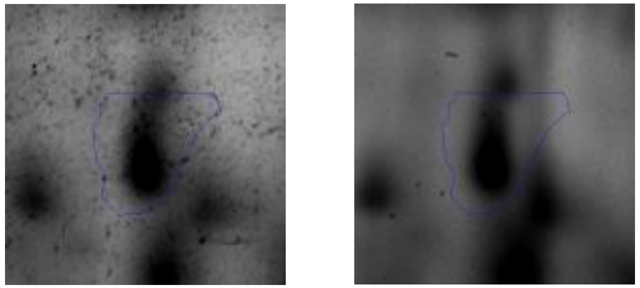

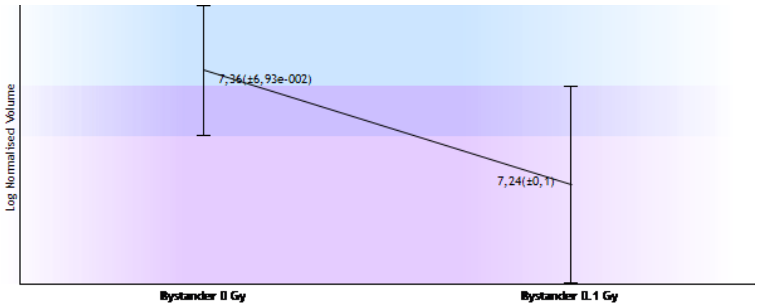

Anova
p = 0,0152

Results WB
CCT3 = -28%

CCT3 (60 kDa)
α- Tubulin (50 Kda)
Gapdh (35 Kda)

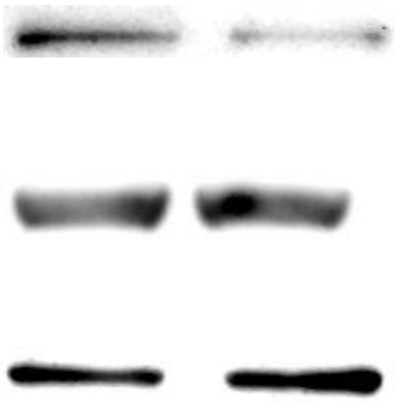

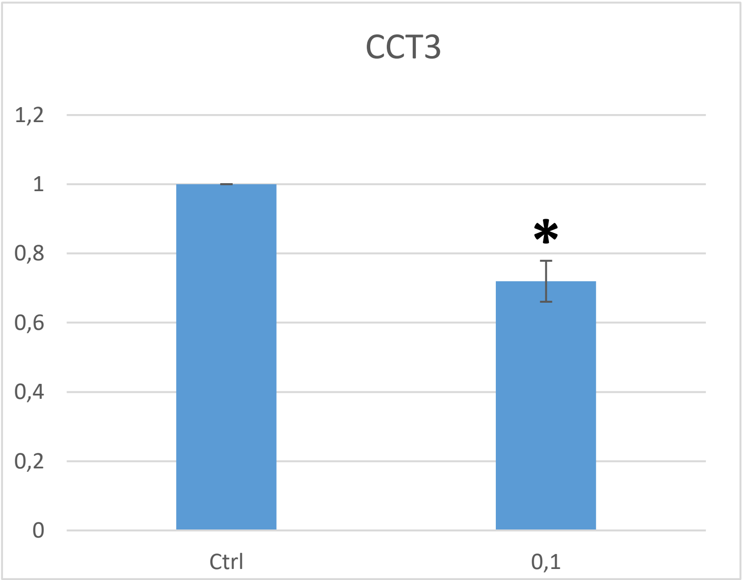

Student test
p = 0,001
